# The Late Founder of "King's" Training School

**Published:** 1915-02-27

**Authors:** 


### The late Founder of "King's" Training School.

BY ONE OF MISS MONK'S NIGHT SISTERS.

Miss K. H. Monk, late matron of King's College Hospital, died at Southampton on February 20, after a sad and tedious illness. She was sixty years of age, and began her nursing career at the age of nineteen, so that a very full share of her life has been devoted to the work that she loved and lived for.

She started in Edinburgh under a "Nightingale" matron and sister in 1874. In 1878 she went to St. Bartholomew's Hospital, London, and she obtained one of the first certificates issued to nurses in the British Isles. For some years she nursed for Sir Thomas Smith, taking a large number of his operation cases, and in 1883 was appointed night sister at Charing Cross Hospital. This work she gave up to take a ward sister's post instead in the same hospital, and had charge of a large female medical ward and the gynæcological ward attached to it. In 1884 she was asked by the council of St. John's House Sisterhood to take the sister-matron's post at King's College Hospital. She at first refused, as she was very loth to give up the actual nursing and her ward sister's post for the administrative work, but the council were very determined to have her, and at last she consented, and became in 1885 sister-matron of "King's" under the committee of management.

Before definitely accepting the matron's post she had been appointed by the War Office (through Lord Hartington) superintendent of a band of nurses for work in the Soudan War; but, of course, this she resigned. She was always interested, however, in military nursing, and was very influential in improving nursing in five of the big military hospitals.

She was a marvellous administrator, and was the founder of the Training School at King's College Hospital. She designed a scheme for giving longer hours off duty than any other hospital, and the material comforts of her nursing staff were always her constant consideration.

In the early days of her administration, rules were conspicuous by their absence. The matron's word, the matron's wish, the matron's point of view was quite sufficient, and the hospital was a very happy training school. The profession was idealised for all who came under her influence, and those who went from her to work as matrons elsewhere carried her views and her rules as their model. Nowhere were the patients' friends treated with greater courtesy. Miss Monk—or Sister Katharine as she was better known—was a pioneer for good in the nursing world, which is certainly the poorer for her loss.

